# Beyond the First Cut: Evaluating LASIK Reoperation Trends

**DOI:** 10.22336/rjo.2025.53

**Published:** 2025

**Authors:** Rajesh Subhash Joshi, Harsha Singh, Riya Rajesh Joshi

**Affiliations:** 1 Department of Ophthalmology, Government Medical College, Nagpur, Maharashtra, India; 2 Department of Ophthalmology, Shri Datta Meghe Institute of Medical College, Nagpur, Maharashtra, India

**Keywords:** laser-assisted in situ keratomileusis, photorefractive keratectomy, flap striae, refractive surgery complications, LASIK = Laser-assisted in situ keratomileusis, SMILE = small incision lenticule extraction, DLK = Diffuse lamellar keratitis

## Abstract

**Aims and Objectives:**

The aim was to evaluate the reoperation rate in laser-assisted in situ keratomileusis surgery (LASIK) and to identify the underlying factors that necessitate retreatment.

**Material and Methods:**

A retrospective study was conducted at a tertiary eye-care centre in central India, which included 288 eyes that underwent LASIK between June 2020 and April 2023, with nine eyes of nine patients who underwent reoperation. Factors included were demographic data, type of reoperation, that is, flap striae correction by its re-lifting, correction of a dislodged corneal flap, clearing debris of diffuse lamellar keratitis, and treatment for over- or under-correction.

**Results:**

Nine out of nine patients underwent reoperation of 288 eyes. The re-surgery rate in our study was 3.12%. Six patients were male, and three were female. The age group of patients was 21-30 years (±2). All patients were from urban areas. The reoperation included re-lifting a dislodged corneal flap (n=3, 1.04%), flap striae correction (n=2, 0.69%), clearing the debris of diffuse lamellar keratitis (n=2, 0.69%), and treatment for over (n=1, 0.35%), or under-correction (n=1,0.35 %).

**Discussion:**

There was no significant gender difference in reoperation rates, although six of the nine cases involved males. This observation warrants further exploration into potential behavioral or occupational factors that might predispose males to flap-related complications.

The retrospective design of this study and its reliance on data from a single surgeon may limit the generalizability of the findings. Multi-center studies with larger sample sizes could provide a more comprehensive understanding of reoperation rates and associated factors. Future research should also explore patient-reported outcomes and satisfaction scores to contextualize the clinical findings better.

Advancements in refractive surgery techniques, such as the adoption of small incision lenticule extraction (SMILE), could also be evaluated for their impact on reoperation rates. Moreover, the role of artificial intelligence in refining patient selection and predicting surgical outcomes represents an exciting avenue for enhancing the safety and efficacy of LASIK procedures.

**Conclusion:**

This study highlights key factors driving LASIK reoperations and emphasizes that addressing complications and leveraging emerging technologies can significantly improve patient outcomes and satisfaction.

## Introduction

Refractive error is the leading cause of visual impairment worldwide [1].

Laser-assisted in situ keratomileusis (LASIK) is a highly popular and effective surgical procedure for correcting refractive errors such as myopia, hyperopia, and astigmatism. With its high precision, rapid visual recovery, and minimal postoperative discomfort, LASIK has become a cornerstone of modern refractive surgery, enhancing visual outcomes and quality of life for millions of individuals worldwide. Advances in laser technology, such as wavefront-guided systems and eye-tracking capabilities, have further improved the safety and efficacy of the procedure, reducing complications and refining outcomes.

Despite its success, LASIK is not without challenges. A subset of patients may require reoperation due to suboptimal visual outcomes or complications. Common reasons for retreatment include under-correction, over-correction, regression of refractive error, residual astigmatism, or patient-reported dissatisfaction with visual quality. Other factors, such as epithelial ingrowth, flap-related issues, and progressive ectasia, can also necessitate re-intervention [2]. The rate of reoperation varies based on factors like patient selection, preoperative assessments, surgical techniques, and postoperative care.

Understanding the incidence and causes of reoperation in LASIK is essential for refining patient selection criteria, improving surgical techniques, and tailoring postoperative management. Furthermore, it is critical to assess how demographic, clinical, and procedural factors contribute to reoperation risk, as this knowledge can guide surgeons in mitigating complications and setting realistic patient expectations.

This study aims to evaluate the reoperation rate in LASIK surgery in a tertiary care setting, with a focus on identifying the underlying factors that necessitate retreatment. By analyzing preoperative profiles, intraoperative parameters, and postoperative outcomes, this research seeks to provide evidence-based insights to enhance patient safety, optimize surgical protocols, and improve overall satisfaction with LASIK surgery.

## Materials and methods

The retrospective record review study was conducted in a tertiary eye care center in central India after obtaining institutional ethics clearance. The institute caters to the various districts within the state and the surrounding states. The study followed the tenets of the Declaration of Helsinki. As a standard practice of the institute, all subjects had given informed written consent for the examination and for the medical records to be used for the approval of research. The records of the patients who underwent refractive surgery between June 2020 and April 2023 were retrieved. The demographic profile, refractive error, spectacle power, refractive workup, type of refractive surgical plan, complications, and follow-up of the patients were recorded. All operative procedures were performed by a single surgeon using the wavefront-guided VISX Star S4 Custom Vue machine (Johnson and Johnson Vision, Santa Ana, CA, USA). Eye tracker and iris registration were used in the treatment of all eyes. Manifest refraction was chosen for the treatment of all eyes.

For LASIK, a corneal flap was created using a microkeratome, lifted to allow excimer laser ablation on the stromal bed, and then repositioned. A bandage contact lens was applied postoperatively, which was removed after 24 hours. Follow-ups were scheduled on days 1, 7, 30, 3, 6, and 12 months postoperatively. The patients were asked to visit as and when required for any problem with the drop in visual acuity or any other problem. Patients who underwent re-surgery within 6 months of the primary surgery were included in the study. Factors studied included the type of intervention performed, which was flap striae correction by its re-lifting, correction of a dislodged corneal flap, clearing debris of diffuse lamellar keratitis, and treatment for over- or under-correction.

For the dislodged corneal flap, the flap was lifted with the help of the flap lifter. The epithelium’s debridement from the flap’s undersurface was performed. Irrigation of the stromal bed was done with a balanced salt solution. The edges of the flap were dried to achieve good adherence. The bandage contact lens was applied. None of the eyes needed a suture to realign the flap.

The statistical analysis was performed using STATA software (v14.0, StataCorp, College Station, TX, USA). Categorical data were described as proportions, while continuous data were presented as mean ± standard error. The data were analyzed by frequency, percentage, Chi-square test, and z-test for testing proportions. A p-value < 0.05 was considered statistically significant.

## Results

The data from 288 eyes from June 2020 to April 2023 were analyzed during the study. Nine eyes of nine patients underwent reoperation, which included re-lifting a dislodged corneal flap (n=3, 1.04%), flap striae correction (n=2, 0.69%), clearing debris of diffuse lamellar keratitis (n=2, 0.69%), and treatment for over (n=1, 0.35%), or under-correction (n=1,0.35 %). The re-surgery rate in our study was 3.12%. Six patients were male, and three were female. The age group of patients was 21-30 years (±2). All patients were from urban areas. **Table 1** summarizes the demographic details of the patients.

**Table 1:** Demographic data

Age	Refractive error (Sphere)	Corneal thickness (Micron)	Dislodged corneal flap	Flap striae	Debris clearance	Overcorrection	Under correction
21-23	-5.5	567	0	1	0	0	0
24-26	-6 (± 2)	524 (± 2)	0	0	2	0	0
27-29	-7 (± 3)	564	1	0	0	1	0
30 and above	-4 (± 2)	522 (± 5)	2	1	0	0	1
Total			3	2	2	1	1

## Discussion

The reoperation rate observed in this study was 3.12%, which is less than the rates reported in the literature. The variability in the reporting of the LASIK reoperation rates is due to differences in the patient selection criteria, surgical technique, and follow-up protocols. In their study, Valdez-García et al. have shown a reoperation rate of 6.85% for all types of treated refractive errors [3]. However, Netto et al. had a high rate of LASIK re-lift procedures (14%) [4]. Mimouni et al. have reported the reoperation rate following hyperopic LASIK as 4.6% [5]. They attributed factors associated with the reoperation to the type of laser used, the microkeratome, and older age. In the extensive series of 21191 cases, Hecht et al. have shown that reoperation was required in 1,234 cases (5.8%) [6]. The relatively low rate reflects advancements in surgical techniques and technologies, such as wavefront-guided systems and precise eye-tracking mechanisms, utilized in this study. These technologies likely contributed to improved surgical accuracy and reduced the need for retreatment.

The most common reasons for reoperation in our cohort were related to flap complications, including flap dislodgement (1.04%) and striae correction (0.69%). These findings highlight the significance of intraoperative precision and postoperative care in preventing flap-related complications. The incidence of flap dislocation in various studies varies from 0.012 to 2.5% [7-10]. Most of the cases of flap dislodgement present during the first week of the LASIK surgery [7,10]. The factors responsible for the flap dislodgement include forceful blinking, minor trauma due to lid squeeze, rubbing of eyes, nasal hinged flap, dry ocular surface, large diameter flap, thin flap, and hyperopic corrections [7-10]. In our study, all the flaps were nasal-hinged, and the thickness of the flap was 130 mm. All the patients had LASIK correction for myopia. Enhanced preoperative counselling and adherence to postoperative instructions, particularly for patients involved in activities with a higher risk of ocular trauma, could further minimize these issues.

The flap striae were observed in two eyes (0.69%). In their multicenter study, Wallerstein et al. have shown that the incidence of flap striae was 0.79% [11]. High preoperative spherical equivalents are associated with a high incidence of flap striae. In our study, one patient had -5.5, and another had -4.00 diopters spherical equivalent.

Diffuse lamellar keratitis (DLK) necessitated reoperation in two cases (0.69%). It is a non-infectious inflammatory condition. The incidence of DLK varies between 0.13% and 18.9% [12,13]. DLK remains a known complication of LASIK, and its prompt recognition and management are crucial for preventing more severe outcomes. Various factors are responsible for the development of DLK. These include old keratome blades, the use of powdered gloves, marking pens, and meibomian gland secretions [14,15]. DLK usually presents within 24-48 hours of the surgery. In our study, both cases presented within 24 hours of the surgery. However, late-onset DLK has also been reported [16]. Its incidence after low-energy femtosecond laser LASIK surgery and small lenticule extraction is rare [17,18]. The use of a standardized postoperative medication protocol and early detection strategies, such as regular slit-lamp examinations, may help reduce the severity of complications.

Under-correction and over-correction accounted for 0.35% of reoperations. The reported incidence of residual refractive error following LASIK is 5-51% [19,20]. Management of correction of residual refractive error includes LASIK enhancement with flap re-lift or PRK [21]. Both cases in our study were treated with photorefractive keratectomy after three months of stabilization of refractive error. These refractive issues highlight the need for meticulous preoperative planning, including accurate measurements of corneal thickness, refractive error, and wavefront aberrations. Surgeons could also benefit from utilizing predictive analytics or nomograms tailored to individual patient profiles to enhance refractive outcomes.

All reoperation cases in this study were among patients aged 21-30 years from urban areas. While the urban demographic may reflect the accessibility of refractive surgery services in such regions, the concentration of cases in this age group could indicate higher expectations for visual outcomes and lower tolerance for residual refractive errors.

There was no significant gender difference in reoperation rates, although six of the nine cases involved males. This observation warrants further exploration into potential behavioral or occupational factors that might predispose males to flap-related complications.

The retrospective design of this study and its reliance on data from a single surgeon may limit the generalizability of the findings. Multi-center studies with larger sample sizes could provide a more comprehensive understanding of reoperation rates and associated factors. Future research should also explore patient-reported outcomes and satisfaction scores to contextualize the clinical findings better.

Advancements in refractive surgery techniques, such as the adoption of small incision lenticule extraction (SMILE), could also be evaluated for their impact on reoperation rates. Moreover, the role of artificial intelligence in refining patient selection and predicting surgical outcomes represents an exciting avenue for enhancing the safety and efficacy of LASIK procedures.

## Conclusion

This study offers valuable insights into the factors contributing to reoperations in LASIK surgery. By addressing the identified complications and incorporating emerging technologies, refractive surgeons can further enhance patient outcomes and satisfaction.
